# Identification and Analysis of Small Interfering RNAs Associated With Heat Stress in Flowering Chinese Cabbage Using High-Throughput Sequencing

**DOI:** 10.3389/fgene.2021.746816

**Published:** 2021-11-01

**Authors:** Waqas Ahmed, Yanshi Xia, Ronghua Li, Hua Zhang, Kadambot H.M Siddique, Peiguo Guo

**Affiliations:** ^1^ International Crop Research Center for Stress Resistance, College of Life Sciences, Guangzhou University, Guangzhou, China; ^2^ Guangzhou Academy of Agricultural Sciences, Guangzhou, China; ^3^ The UWA Institute of Agriculture, School of Agriculture and Environment, The University of Western Australia, Perth, WA, Australia

**Keywords:** brassica, non-coding RNA, high-throughput sequencing, flowering Chinese cabbage, siRNA, heat stress

## Abstract

Endogenous small interfering RNAs (siRNAs) are substantial gene regulators in eukaryotes and play key functions in plant development and stress tolerance. Among environmental factors, heat is serious abiotic stress that severely influences the productivity and quality of flowering Chinese cabbage (*Brassica campestris* L. ssp. *chinensis* var. *utilis* Tsen et Lee). However, how siRNAs are involved in regulating gene expression during heat stress is not fully understood in flowering Chinese cabbage. Combining bioinformatical and next-generation sequencing approaches, we identified heat-responsive siRNAs in four small RNA libraries of flowering Chinese cabbage using leaves collected at 0, 1, 6, and 12 h after a 38°C heat-stress treatment; 536, 816, and 829 siRNAs exhibited substantial differential expression at 1, 6, and 12 h, respectively. Seventy-five upregulated and 69 downregulated differentially expressed siRNAs (DE-siRNAs) were common for the three time points of heat stress. We identified 795 target genes of DE-siRNAs, including *serine/threonine-protein kinase SRK2I*, *CTR1-like*, *disease resistance protein RML1A-like*, and *RPP*1, which may play a role in regulating heat tolerance. Gene ontology showed that predictive targets of DE-siRNAs may have key roles in the positive regulation of biological processes, organismal processes, responses to temperature stimulus, signaling, and growth and development. These novel results contribute to further understanding how siRNAs modulate the expression of their target genes to control heat tolerance in flowering Chinese cabbage.

## Introduction

Environmental stresses such as salinity, drought, and heat can impede crop productivity and even cause plant death (Bita and Gerats, 2013). Flowering Chinese cabbage (*Brassica* campestris L. ssp. chinensis var. utilis Tsen et Lee) generally grows throughout China to meet vegetable demands by consumers (Chen et al., 2017). Nutritional value and leaf freshness are the two major criteria for assessing the quality of fresh vegetables. Long-term yield records and comprehensive analyses of environmental stresses show that high temperatures seriously affect the production and quality of flowering Chinese cabbage. Recent studies have revealed that noncoding RNAs might have significant functions in modulating plant responses to environmental factors (biotic and abiotic stresses) by regulating gene expression during transcriptional and post-transcriptional processes (Srivastava et al., 2017; Wang et al., 2017; Ahmed et al., 2020b).

These molecules are classified into different types depending on their function and synthesis: small interfering RNAs (siRNAs), microRNAs (miRNAs), and long noncoding RNAs (Shukla et al., 2008; Khraiwesh et al., 2012; Zhu et al., 2015). siRNAs and miRNAs are usually 21–24 nt in length, highly conserved, and involved in modulating gene expression in various plant species (Khraiwesh et al., 2012; Yu et al., 2019), including vegetables. In plants, siRNAs are classified into various groups: natural antisense transcript-derived siRNAs, long siRNAs, trans-acting siRNAs, heterochromatic siRNAs, secondary transitive siRNAs, and repeat-associated siRNAs (Khraiwesh et al., 2012; Zhang et al., 2012). siRNAs play an essential role in various biological processes in plants, including abiotic and biotic stress responses (Zhang et al., 2012; Zhong et al., 2013), gene silencing (Kasai et al., 2013), and hybrid vigor (Barber et al., 2012). The Copia-type retrotransposon ONSEN activated in response to heat stress in siRNA biogenesis *Arabidopsis* mutants, indicating the possible involvement of siRNAs in controlling plant responses to heat stress (Ito et al., 2011). Further analysis revealed a regulation mechanism of ONSEN *via* siRNAs in heat responses, suggesting that ONSEN has conserved transcriptional activation facilitated by environmental heat stress in some Brassicaceae species (Ito et al., 2013). Transcript levels of HEAT-INDUCED TAS1 TARGET1 (HTT1) and HTT2 were significantly upregulated under heat stress and targeted by TAS1-derived siRNAs in *Arabidopsis* (Li et al., 2014).

Recent studies showed the involvement of siRNA in seed development and demonstrated that siRNAs could move from maternal seed coats into filial tissues to establish DNA methylation in the next generation (Grover et al., 2020). Besides this, endogenous activated siRNAs in virus-infected Brassicaceae plants exhibit a shared host gene-silencing pattern affecting the stress response and photosynthesis (Leonetti et al., 2021). In cotton, four tas3-siRNAs potentially promote somatic embryogenesis by targeting two ARF genes (Yang et al., 2013). In citrus, 459 differentially expressed siRNAs (DE-siRNAs) reportedly target genes involved in cell differentiation, biological processes, and stress responses (Wu et al., 2015). Only a few studies have revealed the functions of siRNAs in heat stress responses in *Brassica* plants. Therefore, it is essential to elucidate the expression pattern and function of siRNAs involved in regulating heat tolerance in flowering Chinese cabbage to increase its productivity.

In earlier studies, we identified express sequence tag-simple sequence repeat (EST-SSR) markers (Chen et al., 2017) and novel and conserved miRNAs in flowering Chinese cabbage genotype, Youlv 501, after 0 (control), 1, 6, and 12 h of heat stress (Ahmed et al., 2019), and compared miRNAs in flowering Chinese cabbage genotypes, Sijiu-19 and Liuye 50, under heat stress (Ahmed et al., 2020a). In the current study, we constructed four sRNA libraries from leaf samples collected under heat-treated and normal temperature (control) experiments to identify novel siRNAs, compare their expression patterns, and identify their possible functions in monitoring heat tolerance in flowering Chinese cabbage.

## Materials and Methods

### Plant Material and Growth Conditions

Seeds of flowering Chinese cabbage genotype 3T-6, kindly provided by the Guangzhou Academy of Agricultural Sciences (Guangzhou, China), were used in this study. *B. campestris* plants were grown at 28/22°C for 14/10 h (day/night) in a greenhouse at Guangzhou University. Plants at the five-leaf stage were transferred to a growth chamber for heat treatment at 38/29°C (14/10 h). Fully expanded leaves were collected at four time points after 0, 1, 6, and 12 h of heat treatment, then immediately frozen in liquid nitrogen and stored at –80°C until RNA extraction.

### Total RNA Extraction, and sRNA Library Construction and Sequencing

Total RNA was isolated from three biological replicates using TRIzol reagent (Invitrogen, Life Technologies) following the manufacturer’s recommendations. An equal amount of RNA from three replicates for each time point was pooled for library construction using Illumina TruSeq Small RNA Preparation Kit according to the manufacturer’s instructions. In brief, RNA 5′- and 3′-adapters were ligated to total RNA, reverse transcription was performed of complementary DNA (cDNA) constructs, and then 6% denaturing polyacrylamide gel electrophoresis was used to isolate fragments of different lengths. The Beijing Genomics Institute (Shenzhen, China) processed the four cDNA libraries using an Illumina HiSeq sequencer following the standard protocol. Raw sequence reads were analyzed using Illumina’s analysis software.

### Identification of Small Interfering RNAs

After removing low-quality reads, oversized insertion tags, and adapter sequences, the remaining small RNA reads were mapped to the *Brassica* database (http://brassicadb.org/brad/); reads with exact matches were used in further analyses after removing miRNAs, small nuclear RNA, small nucleolar RNA, ribosomal RNA, and transfer RNA sequences. siRNAs were predicted in the sample based on the following criteria: i) match sequences had more than five reads per sample, and ii) the sequences had two overhanging bases that were complemented.

### Analysis of Differentially Expressed Small Interfering RNAs

The siRNA expression of the heat-treated and control samples was compared to determine the siRNA differential expression. DE-siRNAs were identified as described elsewhere (Ge et al., 2017). Briefly, read numbers were normalized as transcripts per million in each library to reflect the siRNA expression. siRNAs with an absolute value of log_2_ ratio  ≥ 1 and *p* < 0.05 were considered DE-siRNAs between the treatments. DE-siRNAs were subjected to hierarchical cluster analysis using the Mfuzz package in R 4.0.5 (Kumar and Futschik, 2007).

### Predicting the Targets of Differentially Expressed Small Interfering RNAs

To identify the target genes of DE-siRNAs, TargetFinder (Xie et al., 2012) and psRobot (Wu et al., 2012) were used as described previously (Ge et al., 2017). Only binding sites commonly predicted by both tools were selected for further analysis to verify the findings and increase the confidence interval.

### Gene Ontology Prediction of Small Interfering RNA-Related Regulatory Pathways

The gene ontology (GO) functional analysis database (http://www.geneontology.org/) was used to predict the key regulatory pathways using a threshold derived from a hypergeometric test with a corrected *p*-value of ≤0.05. GO allocated and classified the query read sequences into different functional groups.

### Verification of Next-Generation Sequencing Results Using RT-qPCR

To verify the siRNA expression data derived from next-generation sequencing, quantitative real-time RT-PCR (RT-qPCR) was performed. We randomly selected 18 siRNAs whose expression was either unchanged, upregulated, or downregulated after heat treatments for RT-qPCR as described earlier (Guo et al., 2007; Guo et al., 2009). The same RNA samples at 0, 1, 6, and 12 h after heat treatments used for sequencing were also used to confirm the expression of DE-siRNAs with RT-qPCR. Briefly, 1 µg of total RNA was reversely transcribed into cDNA using HiScript II 1st Strand cDNA Synthesis Kit (Vazyme Biotech). qRT-PCR was conducted using ChamQ SYBR qPCR master mix (Vazyme, Nanjing, China) in a CFX96 qPCR machine (BioRad, United States) following the manufacturer’s instructions. Specific primers used for RT-qPCR are listed in Supplementary Table 6. 5S rRNA was used as the internal control, and 2^−ΔΔCT^ method was used to determine the siRNAs relative expression level at each time point (Livak and Schmittgen, 2001).

## Results

### Classification of sRNA Sequences

**FIGURE 1:**
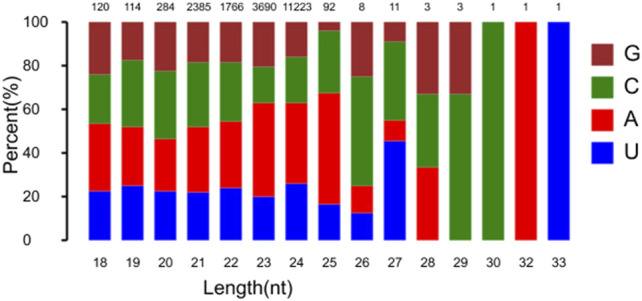
Length distribution of predicted siRNAs in flowering Chinese cabbage. Statistics of first base of all kinds of predicted siRNAs is shown. *X*-axis is length of siRNA in bp; numbers on top are mean numbers of predicted siRNAs.

Sequencing analysis of the constructed RNA libraries generated 28,294,247, 29,924,277, 27,763,656, and 28,729,621 reads after 0, 1, 6 and 12 h of heat stress, respectively. The sequence data were deposited into the National Center for Biotechnology Information Sequence Read Archive database under accession number PRJNA758034. Removal of short valid length, invalid adapter and low-quality reads resulted in 25,939,459, 28,119,719, 25,039,056, and 25,882,948 reads after 0, 1, 6 and 12 h of heat stress, respectively. Detailed information on the reads in each RNA sequence is in Supplementary Table 1. The siRNA length distribution ranged from 20 to 24 nucleotides (nt), with 21 nt as the most common, followed by 24, 22, and 23 nt. Most of the 22–24-nt small RNAs started with C. The highest proportion of nucleotides in the sequence of endogenous small RNAs was occupied by C and A (Figure 1).

### Expressed Small Interfering RNA in Response to Heat Stress

**FIGURE 2:**
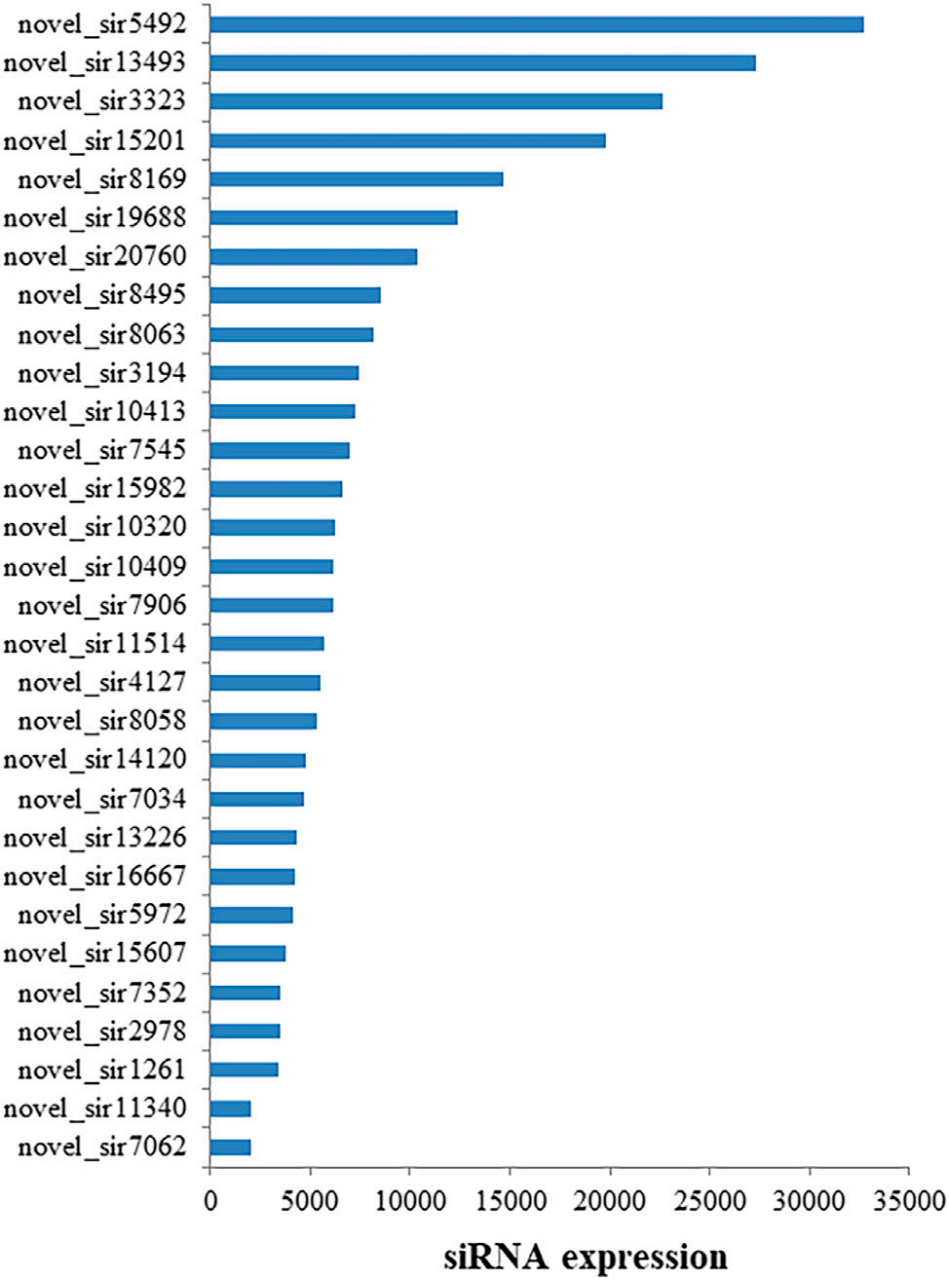
Abundantly expressed siRNAs in flowering Chinese cabbage. siRNA expression is shown in transcripts per million (TPM).

To examine if siRNA is involved in heat tolerance of flowering Chinese cabbage, normalized sequence reads with read counts <30 were excluded from further analysis. Thirty siRNAs showed the high expression in the flowering Chinese cabbage after 0, 1, 6, and 12 h of heat treatments (Supplementary Table 2), of which the novel_sir20760, novel_sir19688, novel_sir8169, novel_sir15201, novel_sir3323, novel_sir13493, and novel_sir5492 showed expression levels of >10,000 followed by novel_sir7062, novel_sir2978, novel_sir14120, novel_sir11514, novel_sir10409, novel_sir10413, novel_sir3194, novel_sir8063, and novel_sir8495 (Figure 2).

### Identification of Differentially Expressed Small Interfering RNAs

**FIGURE 3:**
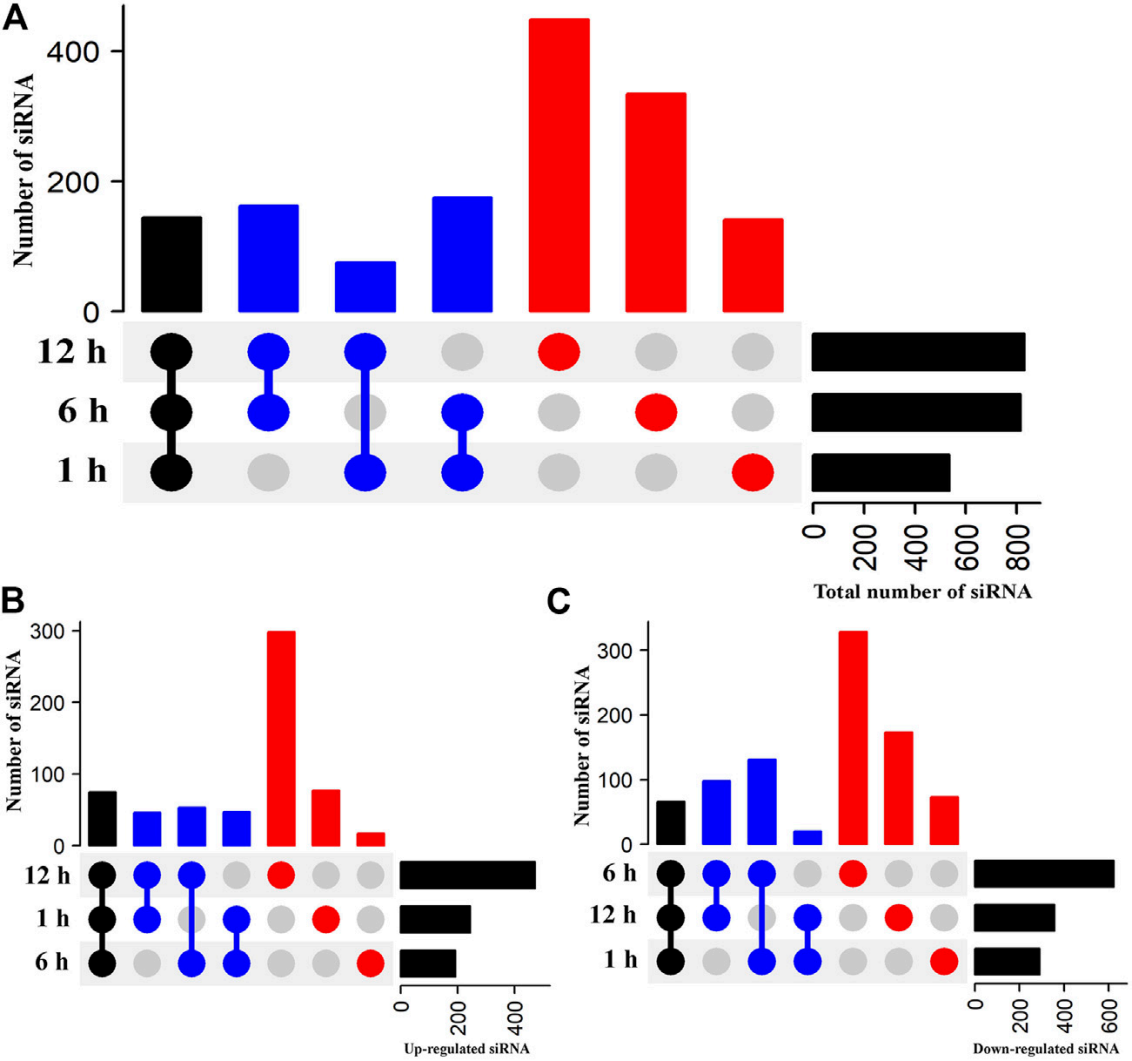
Heat-stress responsive DE-siRNAs in flowering Chinese cabbage. UpSetR plots represent DE-siRNAs after 1, 6, and 12 h of heat stress, relative to control (0 h). Black vertical bars highlight common DE-siRNAs at all three time points, blue vertical bars represent common DE-siRNAs at two time points (e.g., 1 and 6 h), and red vertical bars represent DE-siRNAs at one time point. Horizontal black bars indicate total number of DE-siRNAs at individual time points. **(A)** Total number of DE-siRNAs, **(B)** upregulated DE-siRNAs, and **(C)** downregulated DE-siRNAs in flowering Chinese cabbage after 1, 6, and 12 h of heat stress.

The expression levels of siRNAs at each time point were compared with the control to identify DE-siRNAs that play an essential role in the response of flowering Chinese cabbage to heat stress. An UpSetR plot showed 536, 816, and 829 siRNAs exhibited differential expression in the libraries of flowering Chinese cabbage after 1, 6, and 12 h of heat treatments, respectively (Figure 3A, Supplementary Table 3). Among them, 245, 192, and 472 siRNAs were upregulated after 1, 6, and 12 h of heat treatments, respectively (Figure 3B). After comparing the siRNA expression profiles at the three time points, 144 DE-siRNAs were identified across all three time points, of which 75 were upregulated and 69 were downregulated in flowering Chinese cabbage across all three time points (Figure 3C). Among the upregulated siRNAs, novel_sir10102, novel_sir17115, novel_sir19717, novel_sir18193, novel_sir5840, and novel_sir9841 had the highest levels of differential expression, whereas novel_sir7562, novel_sir21032, novel_sir16370, novel_sir6523, and novel_sir8892 were highly downregulated, suggesting the possible role of siRNAs in heat tolerance of flowering Chinese cabbage (Supplementary Table 3).

**FIGURE 4:**
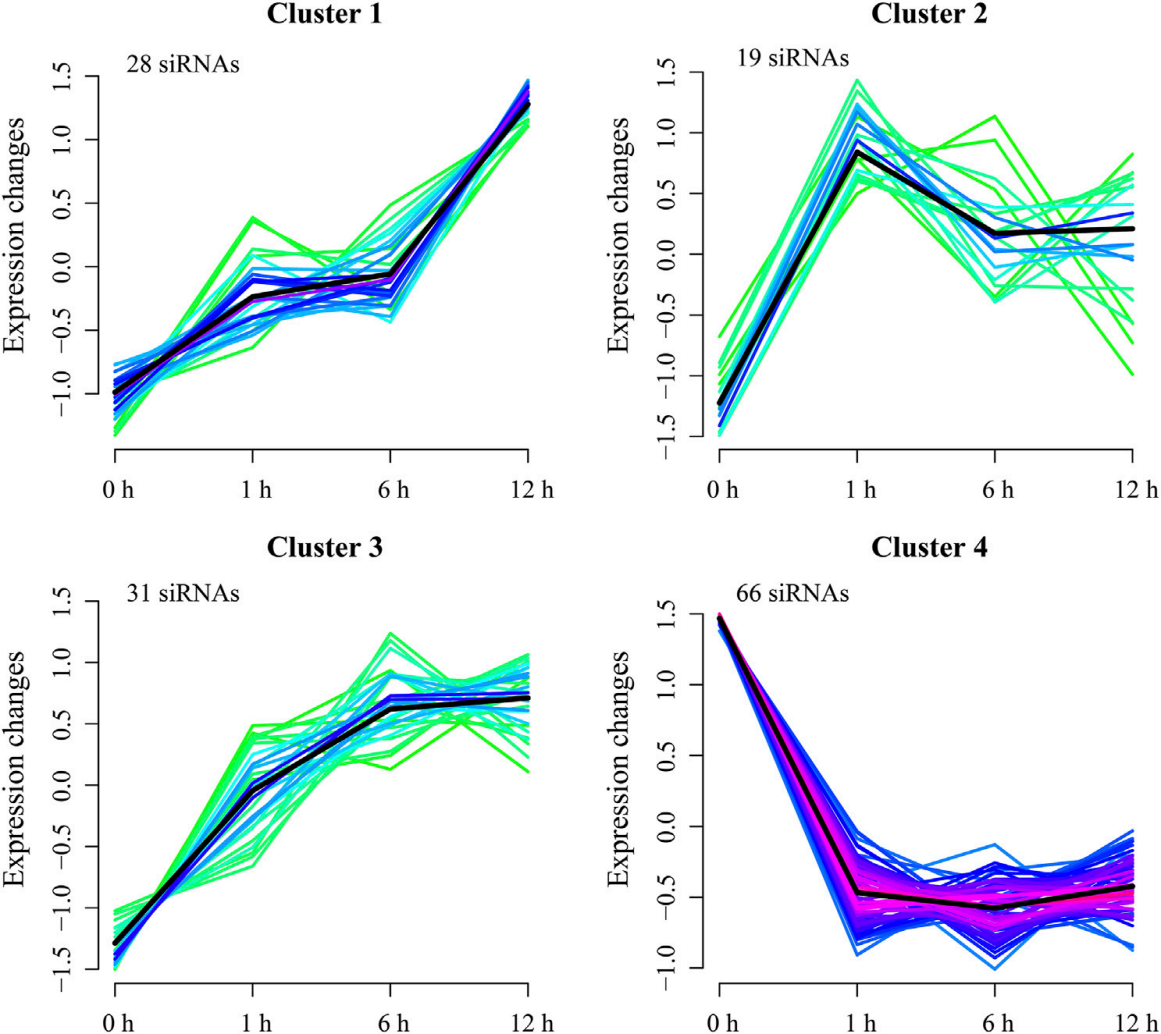
Cluster analysis of DE-siRNAs. Lines reflect expression changes for each DE-siRNA at 1, 6, and 12 h. Black lines are mean changes in expression of siRNAs in each cluster.

Furthermore, cluster analysis separated the DE-siRNAs into four clusters (Figure 4). Cluster 1 consists of 28 upregulated DE-siRNAs at 1, 6, and 12 h of heat treatments, with the maximum expression changes at 6 and 12 h of heat treatments. Cluster 2 has 19 DE-siRNAs that were progressively downregulated at 1 and 6 h and upregulated at 12 h of heat treatments. Cluster 3 contains 31 DE-siRNAs that were upregulated at 1, 6, and 12 h of heat treatments. There are 66 DE-siRNAs in cluster 4 that were downregulated at 1, 6, and 12 h of heat treatments.

### Target Genes of the Differentially Expressed Small Interfering RNAs

**FIGURE 5:**
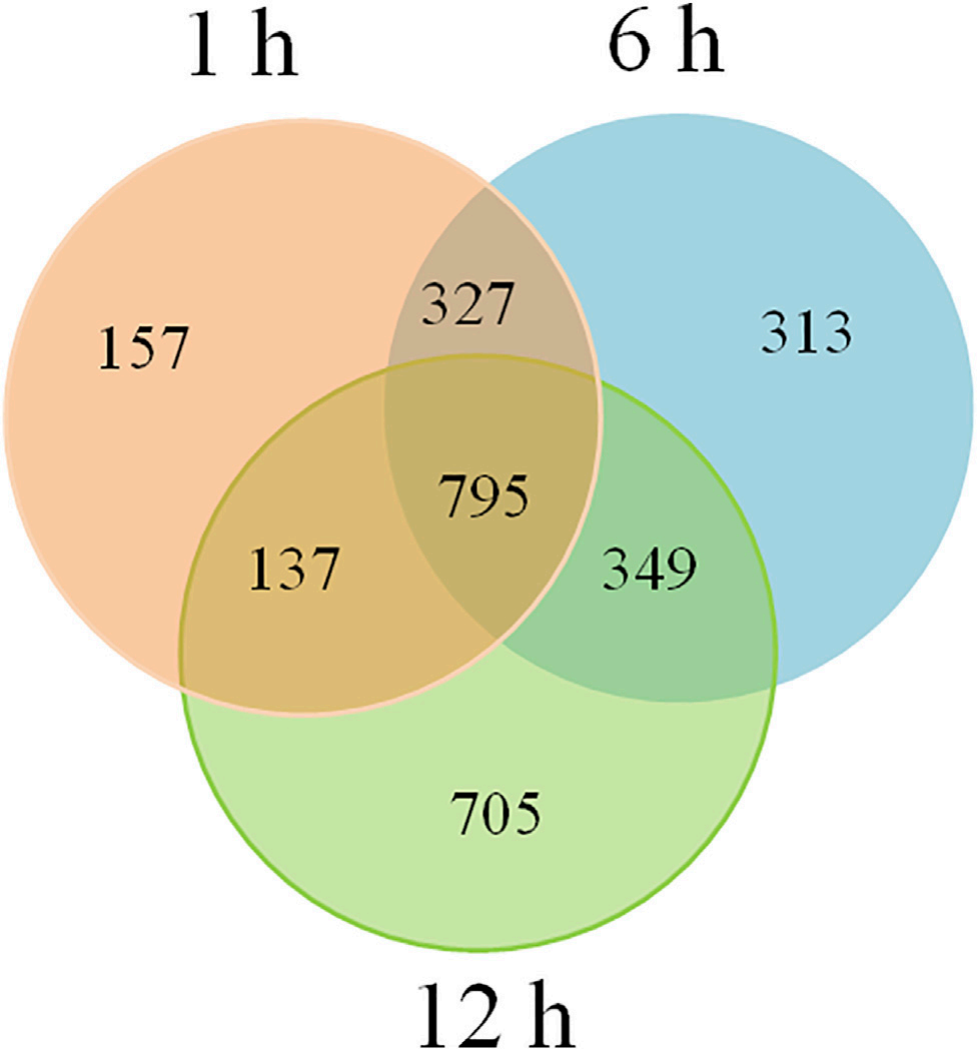
Potential target genes of DE-siRNAs in flowering Chinese cabbage under heat stress.

To investigate the involvement of siRNAs in heat tolerance of flowering Chinese cabbage, we identified 1,415, 1,784, and 1,986 putative target genes of DE-siRNAs for 1, 6, and 12 h of heat treatments, respectively, using psRobot and targetFinder. Among them, 327 target genes were common in the 1 and 6-h heat treatments, 137 target genes were common for 1 and 12 h, 349 target genes were common for 6 and 12 h, and 795 target genes were common for all three time points (Figure 5, Supplementary Table 4). Among targets of DE-siRNAs, 683 (79 upregulated and 604 downregulated), 932 (525 upregulated and 407 downregulated), and 1,077 (721 upregulated and 356 downregulated) were differentially expressed for 1, 6, and 12 h of heat treatments, respectively. Among the heat-induced common target genes of DE-siRNAs, 351 (195 upregulated and 156 downregulated) were differentially expressed in flowering Chinese cabbage. A correlation (*r* ^2^ = 0.90) was observed between DE-siRNAs and differentially expressed target genes of DE-siRNAs (Supplementary Table 4), confirming that the DE-siRNAs identified by next-generation sequencing are real. The target genes include these to encode a disease resistance protein RPS6-like, a putative disease resistance protein At5g66900, a putative LRR receptor-like serine/threonine-protein kinase At1g51860, a disease resistance protein RML1A-like isoform X2, a transcription factor SCREAM2-like protein, a putative disease resistance protein RPP1 isoform X1, a serine/threonine-protein kinase SRK2I, a disease resistance protein TAO1-like isoform X2, a disease resistance protein TAO1-like isoform X4, an RRP12-like protein, a serine/threonine-protein kinase CTR1-like, and a receptor-like protein 12 (Table 1).

**TABLE 1:** Potential target genes of differentially expressed novel siRNAs in flowering Chinese cabbage under heat stress.

miRNA	Target name	Target id	Putative function of target
novel_sir9784	BraA03g010330.3C	XP_009131879.1	putative fatty acyl-CoA reductase 7 isoform X2
novel_sir1979	BraA03g004600.3C	XP_018512591.1	probable disease resistance protein RPP1
novel_sir6288	BraA09g039380.3C	XP_009115492.1	serine/arginine-rich splicing factor RSZ21 isoform X1
novel_sir5666	BraA08g021430.3C	XP_009109360.2	Disease resistance protein TAO1-like isoform X2
novel_sir7548	BraA03g009600.3C	XP_009131792.1	Disease resistance protein TAO1-like isoform X1
novel_sir733	BraA09g015010.3C	XP_013661567.1	Disease resistance protein RML1A-like
novel_sir6315	BraA08g023800.3C	XP_009109657.1	GDSL esterase/lipase At1g29660
novel_sir4946	BraA02g013230.3C	XP_009126995.1	Pectinesterase QRT1-like
novel_sir5161	BraA03g040130.3C	XP_009135779.1	Phospholipase D gamma 1-like
novel_sir9463	BraA05g021940.3C	XP_009102824.1	probable LRR receptor-like serine/threonine-protein kinase At1g29720 isoform X3

### Functional Annotation of Small Interfering RNA Target Genes

**FIGURE 6:**
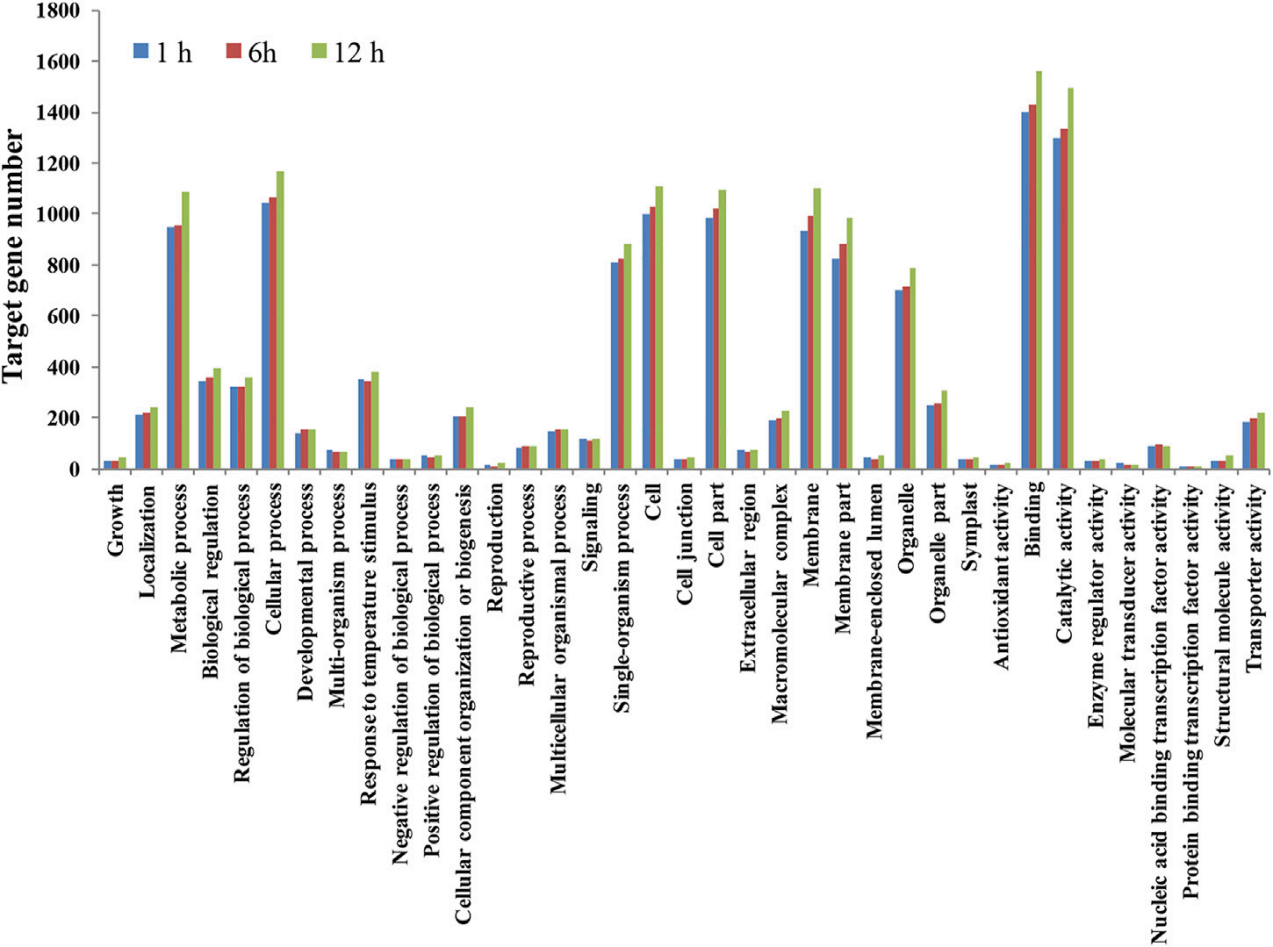
Gene ontology analysis of predicted target genes of heat-stress responsive siRNAs in flowering Chinese cabbage libraries.

GO analysis further classified predicted target genes into three categories based on their functions: biological process, molecular function, and cellular component (Figure 6; Supplementary Table 5). In the biological process category, enriched GO terms included a response to temperature stimulus, positive regulation of the biological process, biological regulation, signaling, developmental process, single organism process, multicellular organismal process, and growth. In the cellular component group, the most enriched GO terms included macromolecular complex, membrane, cell, extracellular region, cell junction, organelle, and organelle part. In the molecular function category, most GO terms included protein binding transcription factor activity, structural molecular activity and transporting activity, binding, enzyme regulator activity, and catalytic activity.

### Quantitative Analysis Verified Next-Generation Sequencing Results

**FIGURE 7:**
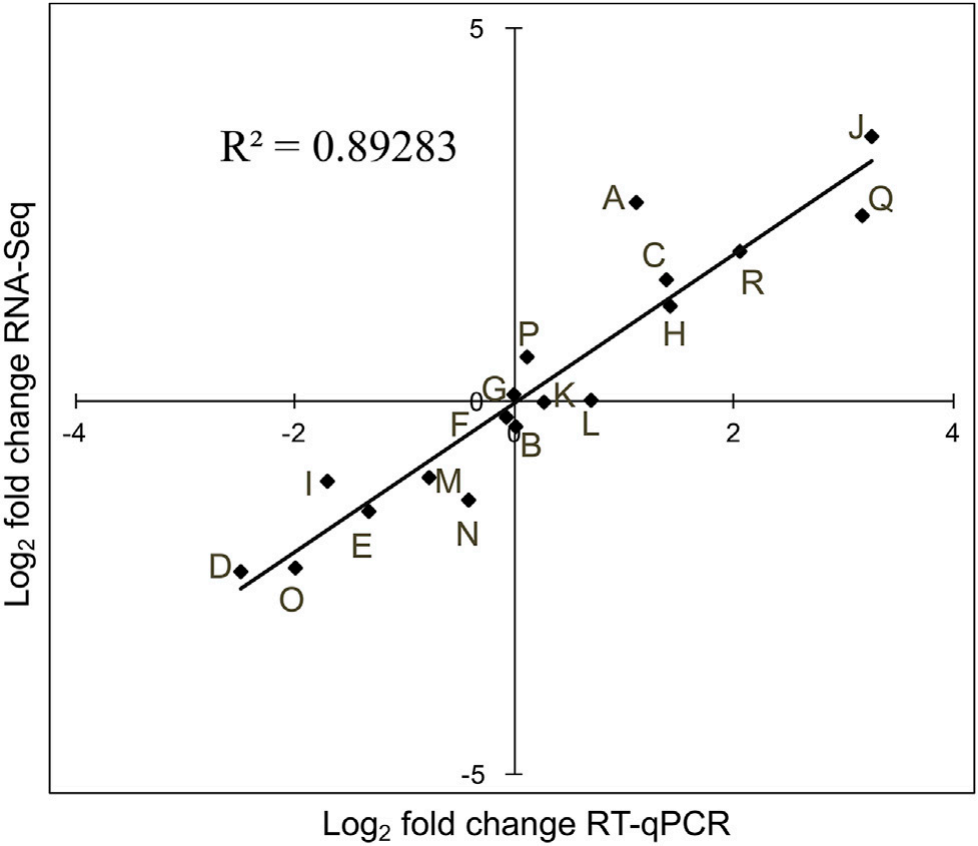
Validation of DE-siRNAs using RT-qPCR. RNA-seq data (*Y*-axis) were plotted against RT-qPCR log_2_-fold change values (*X*-axis). Letters A to R represent siRNAs, being novel_sir966, novel_sir5526, novel_sir6288, novel_sir7548, novel_sir4946, novel_sir6523, novel_sir7091, novel_sir5666, novel_sir6315, novel_sir9784, novel_sir6347, novel_sir4575, novel_sir9463, novel_sir5161, novel_sir733, novel_sir8591, novel_sir3609, and novel_sir1979, respectively.

To validate the differential expression of the siRNAs identified by next-generation sequencing, the expression levels of 18 siRNAs, representing unchanged, upregulated, or downregulated expression, were measured by RT-qPCR. A high correlation (*r* ^2^ = 0.892) was observed between sequencing data and RT-qPCR (Figure 7), confirming that the DE-siRNAs identified by next-generation sequencing are real.

## Discussion

Plants have established robust and sophisticated mechanisms to cope with biotic and abiotic stresses for adaptive growth responses, such as reestablishing and restoring cellular homeostasis (Wang et al., 2004; Mittler, 2006). Vegetable plants respond to external stimuli through well-defined reprogramming and specific orchestration of gene expression regulation of transcriptional activities to minimize the stimuli’s effect on their physiological states (Dresselhaus and Hückelhoven, 2018). However, despite these regulatory mechanisms, environmental factors play key roles in regulating plant growth, survival, and yield. Heat stress tolerance is a quantitative trait, and its expression can involve the fine regulation of stress-related genes in plants (Golldack et al., 2014; Suzuki et al., 2014). Recent advances in next-generation sequencing have identified and functionally characterized many genes involved in responses to environmental stresses, signifying their functions in the maintenance of stress tolerance (Ahmed et al., 2020b). siRNAs are involved in regulating abiotic stress responses in plants, but limited information is available on their function in response to heat stress. In the present study, we identified DE-siRNAs and potential target genes that might play key roles in regulating heat tolerance in the flowering Chinese cabbage 3T-6 genotype. Most of the identified siRNAs ranged from 20 to 24 nt, with 291, 624, and 357 downregulated and 245, 192, and 472 upregulated after 1, 6, and 12 h of heat treatment, respectively. Likewise, Ge et al. (2017) identified the same sized siRNAs associated with maize embryonic callus formation. Yao et al. (2010) revealed three siRNAs in wheat with significantly upregulated expression levels under cold stress and significantly downregulated levels under heat stress. *ONSEN*, a *copia*-type retrotransposon, was transcriptionally active under heat stress in *Arabidopsis* seedlings (Ito et al., 2011). High accumulation of *ONSEN* transcripts was detected in the progeny of heat-stressed plants deficient in siRNAs, suggesting that *ONSEN* activation is controlled by cell-specific regulatory mechanisms (Matsunaga et al., 2012). Phylogenetic analysis of the *ONSEN* transposon family across species of Brassicaceae revealed that conservation of *ONSEN* sequences is closely related and together comprise a clade, an important finding for understanding evolutionary adaptation to thermal environmental stress (Ito et al., 2013). The conserved preferential insertion and heat activation near genic regions among species of Brassicaceae indicate that *ONSEN* could alter gene regulatory networks following heat stress (Ito et al., 2013).

A high correlation coefficient (*r* ^2^ = 0.892) between RT-qPCR and next-generation sequencing data indicated the consistent differential expression profiles (up/downregulation) between RT-qPCR and next-generation sequencing data, which agrees with previous studies (Xia et al., 2014; Yao et al., 2016). Likewise, in a previous study, 778, 1,075, and 652 siRNAs and 326, 172, and 1,832 siRNAs were significantly downregulated and upregulated between stages I and II, stages I and III, and stages II and III, respectively, indicating that these siRNAs might have vital roles in inducing immature embryonic calli (Ge et al., 2017). Furthermore, in rice, several siRNAs (P86-H10, P91-A10, P88-A8, and P8-E2) were ubiquitously expressed, whereas others (P65-B7, P94-H11, P104-G7, P108-D3, and P103-B2) were preferentially expressed in the inflorescence, suggesting that endogenous siRNAs are differentially expressed in developmental stages and different tissues (Sunkar et al., 2005). Deep sequencing of siRNAs in flower tissues of WT and nrpd1a and nrpd1b mutants of Arabidopsis identified more than 4,200 loci that produced siRNAs in a PolIV-dependent manner, with PolIVb reinforcing siRNA production by PolIVa (Mosher et al., 2008). Sunkar et al. (2005) identified 284 unique putative siRNA sequences corresponding to 942 genomic loci in a tissue-specific or the developmental-stage-specific manner in *Arabidopsis*. These identified targets established evidence for both cis-silencing and trans-silencing of the target messenger RNAs by siRNA-guided cleavage, with most of these siRNAs (225 of 284) involved in developmental processes and/or active meristematic/cell division. In the current study, DE-siRNAs were clustered into four clusters based on their expression patterns. Likewise, the role of siRNAs was characterized under drought stress in rice, siRNAs that exhibit up/downregulated expression are highly conserved and clustered, indicating that siRNAs are mainly responsible for drought-related damage to plants as the drought stress continues and their functional roles are related to well-known drought-related pathways (Jung et al., 2016).

In maize, 576 potential target genes of DE-siRNAs that regulated embryonic callus formation were identified, and most could play key roles in anion channel activity, binding, protein dimerization activity, chaperone binding, and gated channel activities (Ge et al., 2017). We identified 795 target genes of DE-siRNAs that might play a key role in heat stress responses. In addition, most of the predictive target genes could regulate responses to temperature stimulus, developmental processes, single organism processes, protein binding transcription factor activity, and the macromolecular complex (Supplementary Table 4).

In conclusion, this study identified a comprehensive dataset of heat-responsive siRNAs for *B. campestris* L. ssp. *chinensis* var. *utilis* Tsen et Lee under heat stress. The siRNA information, their differential expression, and identified messenger RNA targets will be useful for the adaptation of flowering Chinese cabbage and close species to future stress encounters, thus enhancing yield stability and crop resilience when breeding this important vegetable crop.

## Data Availability

The datasets presented in this study can be found in online repositories. The names of the repository/repositories and accession number(s) can be found below: https://www.ncbi.nlm.nih.gov/, PRJNA758034.
